# Medial Epicondylitis: A Review of Clinical Presentation, Diagnosis, and Management in the United Kingdom

**DOI:** 10.7759/cureus.102264

**Published:** 2026-01-25

**Authors:** Abdullah Tahir, Nihal Chanian, Simrat Tiwana, Muhammad Arham Sahu, John Blackwell

**Affiliations:** 1 Trauma and Orthopaedics, University Hospitals of North Midlands NHS Trust, Stoke-on-Trent, GBR; 2 Radiology, Royal Wolverhampton NHS Trust, Wolverhampton, GBR; 3 Trauma and Orthopaedics, University Hospitals of Leicester NHS Trust, Leicester, GBR; 4 Trauma and Orthopaedics, Royal Cornwall Hospitals NHS Trust, Truro, GBR; 5 Trauma and Orthopaedics, Walsall Healthcare NHS Trust, Walsall, GBR

**Keywords:** baseball elbow, elbow pain, forehand tennis elbow, golfer’s elbow, medial epicondylitis, suitcase elbow, thrower’s elbow

## Abstract

Medial epicondylitis is a chronic tendinopathic disorder involving the flexor-pronator origin of the medial elbow, characterised by pain and functional limitation related to repetitive loading and microtrauma. The condition exemplifies the broader spectrum of overuse injuries that result from cumulative strain exceeding the capacity for tendon repair. Diagnosis relies primarily on clinical evaluation, with imaging modalities used selectively to define the extent of pathology or to exclude alternative causes of medial elbow pain. Management follows a structured, stepwise approach, centred on conservative therapy, including rest, physiotherapy, and graduated strengthening programmes that are supported by adjunctive interventions when symptoms persist. Surgical management is considered only in cases of chronic, treatment-resistant disease. Growing recognition of this condition in both community and inpatient populations highlights its relevance to multidisciplinary care and underscores the value of coordinated management strategies that combine clinical assessment, rehabilitation, and targeted use of imaging to optimise outcomes.

## Introduction and background

Epicondylitis is a common cause of elbow pain among athletes and the general population, affecting both medial and lateral epicondyles of the elbow, with lateral epicondylitis being significantly more prevalent [1]. Medial epicondylitis, commonly known as golfer’s elbow, presents as chronic tendinosis of the common flexor-pronator origin at the medial epicondyle of the humerus, commonly due to overuse or repetitive stress [2].

Medial epicondylitis has traditionally been regarded as a sports-related condition, managed in community-based settings, with hospital care being reserved for complex or refractory cases. However, patients admitted to hospital for unrelated conditions often develop, or experience exacerbation of, tendinopathies secondary to immobilisation, deconditioning, or altered biomechanics. Hospital physicians frequently encounter these presentations during inpatient rehabilitation, post-operative recovery, or occupational health assessments. Awareness of medial epicondylitis in these contexts allows for earlier intervention, reducing morbidity and facilitating timely discharge.

## Review

Anatomy and pathophysiology

**Figure 1:**
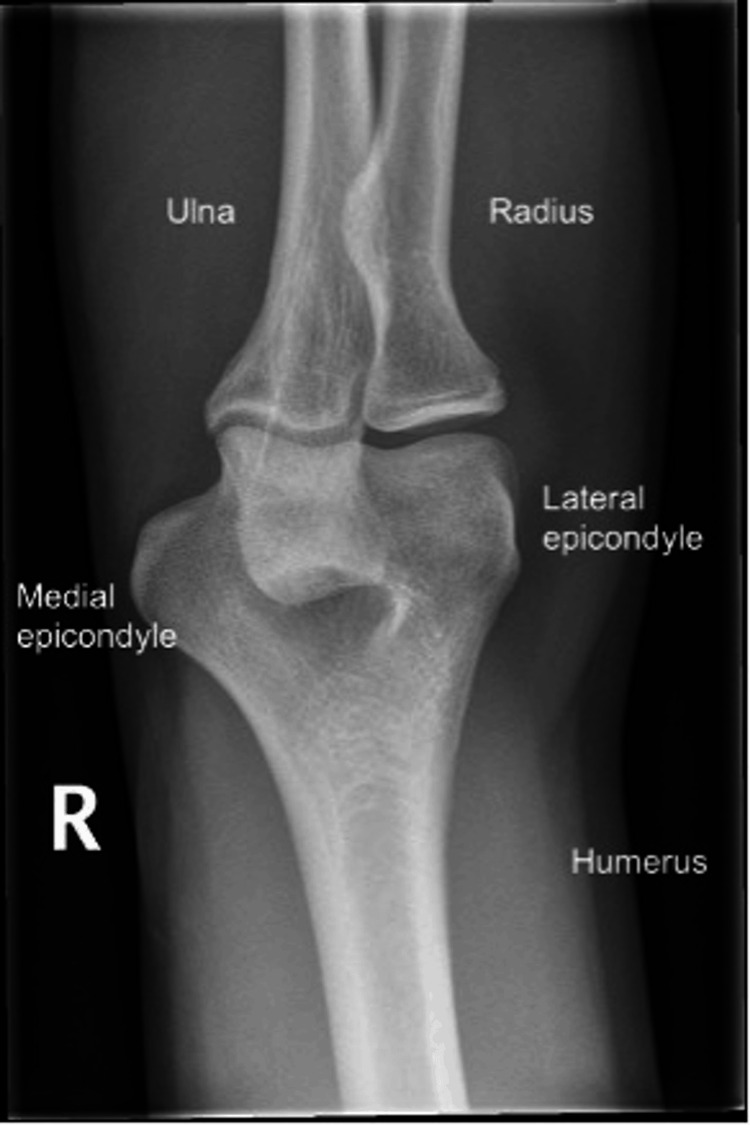
Anterior-posterior view of the right elbow (normal) Credit: [4]

The medial epicondyle is the medial prominence of the distal humerus. It is the origin of the common flexor tendon, which anchors the pronator teres, flexor carpi radialis, palmaris longus, flexor digitorum superficialis, and flexor carpi ulnaris muscles [3]. The ulnar collateral ligament also arises here, contributing to elbow stability. The ulnar nerve originates from the brachial plexus and travels down the arm, passing posteriorly to the medial epicondyle, explaining the frequent coexistence of neuropathic symptoms. Figure 1 demonstrates an anterior-posterior radiographic view of a normal right elbow.

Medial epicondylitis, or ‘golfer’s elbow,’ - similar to the more common lateral epicondylitis, or ‘tennis elbow’ - is a condition common among athletes. Medial epicondylitis is a result of repetitive stress injury at the origin of the common flexor tendon. It is an overuse injury often associated with golfing, throwing, and racquet sports, including tennis and badminton [5]. Repetitive motions, improper technique, and excessive force are contributing factors to inappropriate stresses at the attachment of the common flexor tendon.

Histopathological studies demonstrate that medial epicondylitis is not an inflammatory disorder, but a degenerative tendinopathy, which is characterised by fibroblast proliferation, collagen disorganisation, and neovascularisation - collectively termed angiofibroblastic hyperplasia [2].

Epidemiology

Epicondylitis is approximated to affect 1%-3% of the population each year [6]. Lateral epicondylitis remains a significantly more common presentation, with medial epicondylitis accounting for 10% of diagnoses [7]. Prevalence is highest among patients aged 40 years and older, with men and women being at equal risk of developing symptoms [6]. Smoking, obesity, and multiple comorbidities are strong determinants of developing epicondylitis [8].

In addition to lifestyle factors, several occupational exposures have been associated with repetitive loading of the flexor-pronator complex, including prolonged gripping, forceful forearm movements and exposure to vibration. These factors are particularly relevant in individuals engaged in manual labour, such as construction workers and factory-based machine operators [6].

Clinical presentation

Medial epicondylitis typically presents with medial elbow pain radiating into the forearm and wrist, aggravated by gripping, wrist flexion, or forearm pronation [8]. Patients may report associated elbow stiffness, weakness, or paraesthesia in the ulnar nerve distribution, reflecting the close anatomical relationship to the ulnar groove.

Symptoms generally develop gradually and without a clear precipitating event, often described as a dull ache, localised to the medial aspect of the elbow. On examination, maximal tenderness is found just distal to the medial epicondyle, at the common flexor tendon origin [2]. Up to 20% of patients demonstrate associated ulnar nerve involvement [8].

Acute presentations may exhibit localised swelling, erythema, or warmth, though these features are uncommon in chronic cases. A thorough history should explore the onset and duration of symptoms, occupational and sporting activities, and aggravating movements.

In hospital practice, these symptoms can mimic or coexist with systemic inflammatory arthropathies, cervical radiculopathy, or drug-induced myopathy. A focussed musculoskeletal examination enables clinics to differentiate medial epicondylitis from more serious pathologies, reducing unnecessary investigations and supporting timely multidisciplinary referral.

Differential diagnoses

Elbow pain is a common presenting symptom to healthcare services. Key considerations include, but are not limited to, ulnar nerve entrapment, ligamentous injuries, olecranon bursitis, arthritis, and referred pain from cervical radiculopathy [9]. A thorough assessment, including history, physical examination, and imaging, if required, is essential to accurately differentiate these conditions.

Examination

Accurate clinical diagnosis of medial epicondylitis requires a structured physical examination to assess for tendinopathy at the affected site, while ruling out other causes of medial elbow pain. Clinicians should first inspect the elbow for signs of swelling, erythema, or deformity, which may suggest a more acute pathology. On palpation, tenderness anterior and distal to the medial epicondyle, corresponding to the flexor-pronator origin, will be most significant in diagnosing medial epicondylitis. 

Examination should include assessment of both active and passive range of motion in both the elbow and wrist joints [10]. This is because range of motion is preserved in medial epicondylitis, allowing exclusion of acute intra-articular pathology. In the active component, the patient’s arm will be positioned in supination and extension at the elbow. They will be asked to perform wrist flexion and forearm pronation against resistance. If positive, this test will evoke pain at the medial epicondyle. Passive assessment of wrist extension will also induce pain. 

Given the overlap in clinical presentation, it may be appropriate to evaluate the integrity of the ulnar collateral ligament of the elbow. This is commonly injured in sports that involve throwing or repetitive motions. The elbow valgus stress test can be performed by applying a valgus force to the elbow while it is flexed to 20 degrees. The clinician should assess for widening of the medial joint line, or pain on the other side [11].

For a comprehensive assessment, it is important to examine the joints above and below the site of pain. Assessment of the neck and shoulder is needed to rule out cervical radiculopathy, shoulder weakness, or referred pain. The Spurling’s test is one of the examinations that can be used for assessment of the cervical spine when looking for cervical radiculopathy [12]. If positive, pain will be elicited down the ipsilateral arm on neck extension, lateral flexion, and axial compression.

Investigations

Medial epicondylitis can normally be diagnosed through history and clinical examination alone; however, when the diagnosis remains uncertain, or patients are unresponsive to therapy, further investigations are warranted.

Phlebotomy and haematological analysis can be useful when inflammatory or autoimmune arthropathies are among the differentials. Nerve conduction studies and electromyography should be considered if there is suspected impingement of the ulnar nerve within the cubital tunnel [13].

Plain radiographs provide poor visualisation of the common flexor tendon and muscles of the elbow; however, they may be useful in demonstrating secondary features of enthesopathy and tendinopathy, such as fragmented cortical avulsion of the medial epicondyle or intratendinous calcification [14].

Musculoskeletal ultrasonography is generally considered the first-line investigation, with a recent study demonstrating high diagnostic sensitivity and specificity (95.2% and 92%, respectively) [15]. Imaging findings include heterogeneous echogenicity of the flexor tendon, with associated intratendinous calcification and reactive oedema. Full tears may be visible as areas of low echogenicity, with tendon discontinuity. Colour Doppler may show reactive hyperaemia in such cases [16].

**Figure 2:**
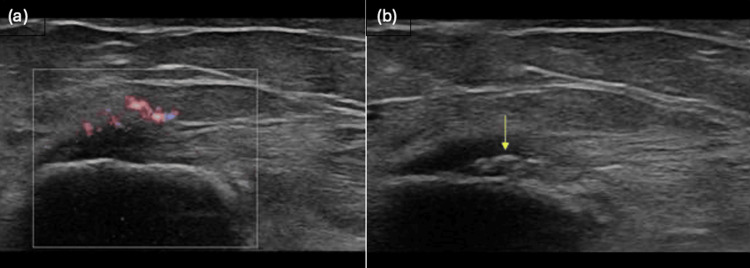
Ultrasound images showing reactive hyperaemia (panel 2a, left) and intratendinous calcification (panel 2b, right) in a patient with medial epicondylitis Credit: [17]

Figure 2a shows reactive hyperaemia in the common flexor-pronator origin on a colour-mode ultrasound image, whilst Figure 2b demonstrates intratendinous calcification, both of which are diagnostic features of medial epicondylitis.

Magnetic resonance imaging (MRI) is the gold standard imaging modality for diagnosing medial epicondylitis; however, it is less readily available and, therefore, only used when symptoms persist despite a normal ultrasound assessment. Features on MRI include regions of increased signal within the common flexor tendon origin on both T1- and T2-weighted fast spin-echo sequences. There may be adjacent soft tissue or bone marrow oedema present [16].

**Figure 3:**
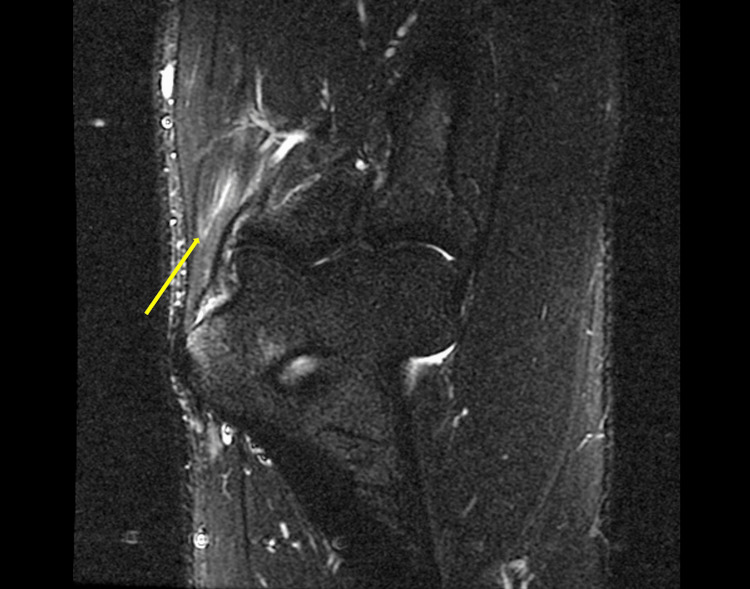
Coronal T2-weighted STIR MRI sequence showing hyperintense signal within the common flexor tendon fibres Credit: [18] STIR, short tau inversion recovery; MRI, magnetic resonance imaging

Figure 3 demonstrates areas of high signal in the common flexor tendon fibres adjacent to the medial epicondyle on a coronal T2-weighted short tau inversion recovery (STIR) MRI sequence, reflecting tendinopathy and reactive oedema.

Management

The primary treatment approach for medial epicondylitis is non-operative management, with an emphasis on rehabilitation of the pathological tendon and on the prevention of disease recurrence. This approach is commonly classified into three phases.

Phase 1 involves relative rest of the common flexor-pronator complex; therefore, activities such as repetitive gripping and forearm pronation are avoided. Ergonomic adjustments in the workplace are recommended to minimise tendon strain. Symptom control includes appropriate analgesia, with topical non-steroidal anti-inflammatory drugs recommended as first-line therapy, alongside adjunctive cryotherapy. Corticosteroid injections may be considered in severe cases refractory to other analgesics; however, their effects are often short-lived and do not provide a long-term solution [19]. Bracing options, including counterforce elbow straps or night splints, may be of some benefit, as load reduction at the tendon origin can help relieve pain [20].

Phase 2 focuses on rehabilitation and strengthening. Eccentric loading exercises actively strengthen the flexor-pronator tendon complex, while proprioceptive training can improve neuromuscular control and enhance joint stability. These modalities are considered particularly effective, with stretching and manual therapy providing complementary benefits [19].

Phase 3 aims to restore function while minimising the risk of recurrence. This involves the gradual reintroduction of task-specific movements, such as gripping, lifting, and throwing, as well as patient education on the importance of warm-up and adequate rest [21]. These phases are summarised in Table 1 [22].

**Table 1:** Phases of non-operative management for medial epicondylitis Source: Adapted from [22]

Phases of Non-operative Management for Medial Epicondylitis
Phase 1 - Pain control and protection
Phase 2 - Rehabilitation and strengthening
Phase 3 - Return to function and prevention

Alternative treatments are increasingly being incorporated into the management of medial epicondylitis. Dry needling involves the insertion of a thin, solid filiform needle into myofascial trigger points, which can reduce muscular tension and result in neuromodulation of pain. Low-energy laser therapy has been shown to manage pain and inflammation by enhancing mitochondrial activity and reducing oxidative stress, thereby stimulating cellular repair [19]. Other adjunctive therapies include ultrasound therapy, iontophoresis, and platelet-rich plasma (PRP) therapy [19,20].

Surgical management may be considered in patients with persistent symptoms despite an extended period of non-operative treatment, typically 6 to 12 months [23], following exclusion of alternative pathological causes. An exception may be made for athletes with definitive tendon disruption identified on MRI. Although surgical techniques vary depending on individual presentation, the core approach involves surgical exploration, debridement, and repair of the pathological tendon tissue, followed by reattachment of the functional flexor-pronator complex [20]. Post-operative care emphasises rest and a progressive rehabilitation programme.

## Conclusions

Medial epicondylitis is a prevalent and functionally limiting tendinopathy that, while traditionally associated with athletic overuse, carries increasing relevance to hospital medicine. It is often a clinical diagnosis, with the use of further investigations reserved for when the diagnosis remains uncertain. Effective management should focus on activity modification, physical therapy, and optimisation of pain management. Operative management can be indicated for patients with persistent symptoms who fail to respond to conservative techniques. Understanding the degenerative nature, risk factors, and response to conservative therapy enables hospital clinicians to deliver effective multidisciplinary care.
